# Supplementary material for: In Situ Formation of Acidic Comonomer during Thermal Treatment of Copolymers of Acrylonitrile and Its Influence on the Cyclization Reaction
Source: Polymers (Basel). 2024 Oct 7;16(19):2833. doi: 10.3390/polym16192833 (PMC11478795; doi:10.3390/polym16192833)

In situ formation of acidic comonomer during thermal treatment of copolymers of acrylonitrile and its influence on the cyclization reaction

Roman V. Toms[1], Daniil A. Ismaylov[1], Alexander Yu. Gervald[1], Nickolay I. Prokopov[1], Anna V. Plutalova[2], XX[3], Elena V. Chernikova[2,4,*]

[1] MIREA – Russian Technological University, Institute of Fine Chemical Technologies named by M.V. Lomonosov, 119571, Av. Vernadskogo, 86, Moscow, Russian Federation
[2]Lomonosov Moscow State University, Faculty of Chemistry, 119991, Lenin Hills, 1, bld.3, Moscow, Russian Federation
[3] Umatex group, Russian Federation, 109316, Volgogradskiy Av., 42, bld. 13, Moscow, Russian Federation
[4] A.V. Topchiev Institute of Petrochemical Synthesis of Russian Academy of Sciences, 119991, Leninsky Av., 29, Moscow, Russian Federation

* Corresponding authors: *chernikova_elena@mail.ru* and *toms.roman@gmail.com*

Table of contents

Figure S1. FTIR spectra of AN–TBA copolymers synthesized from different monomer feeds before and after thermal treatment of their films at 140 °C for required period, specified on the Figures: f_{TBA} = 5.0 (a), 7.5 (b), and 10.0 mol. % (c). 2

Figure S2. FTIR spectra of AN–TBA copolymers synthesized from different monomer feeds before and after thermal treatment of their films at 160 °C for required period, specified on the Figures: f_{TBA} = 5.0 (a), 7.5 (b), and 10.0 mol. % (c). 3

Figure S3. DSC thermograms recorded in argon of copolymers of AN–TBA with various content of TBA synthesized using various amounts of ME. (a) 1 mol. % TBA and 0.05 mol. % ME; heating rate 5 (1), 10 (2), and 20 °C/min (3); (b) 2.5 mol. % TBA and 0.3 mol. % ME; heating rate 5 (1), 10 (2), and 20 °C/min (3); (c) 5 mol. % TBA and 0.3 mol. % ME; heating rate 2.5 (1), 5 (2), 10 (3), and 20 °C/min (4); (d) 10 mol. % TBA and 0.05 mol. % ME; heating rate 2.5 (1), 5 (2), and 10 °C/min (3). 4

Figure S4. Dependences of $\ln\varphi/Tp2$ on $1/T_p$ obtained from DSC thermograms of copolymers of AN and TBA at various heating rates; f_{TBA} = 1 (1), 2.5 (2, 3), 5 (4, 5), 7.5 (6, 7), and 10 mol. % (8, 9); peak II (2, 4, 6, 8), peak III (1, 3, 5, 7, 9). 5

Figure S5. FTIR spectra of the films of AN–TBA copolymers subjected to thermal treatment at 200 (a, c, e) and 225 °C (b, d, f) in argon at various times (indicated on Figure): f_{TBA} = 1 (a, b), 2.5 (c, d), and 5 mol. % (e, f). 6

Figure S6. FTIR spectra of the films of AN–TBA copolymers subjected to thermal treatment at 200 (a, c, e) and 225 °C (b, d, f) in air atmosphere at various times (indicated on Figure): f_{TBA} = 1 (a, b), 2.5 (c, d), and 5 mol. % (e, f). 8

Figure S7. FTIR spectra of the films of AN–BA–TBA copolymers subjected to thermal treatment at 200 (a, c) and 225 °C (b, d) in argon at various times (indicated on Figures): f_{TBA} = 1.0 (a, b) and 2.5 mol. % (c, d). 10

Figure S8. FTIR spectra of the films of AN–BA–TBA copolymers subjected to thermal treatment at 200 (a, c) and 225 °C (b, d) in air atmosphere at various times (indicated on Figures): f_{TBA} = 1.0 (a, b) and 2.5 mol. % (c, d). 11

Figure S1. FTIR spectra of AN–TBA copolymers synthesized from different monomer feeds before and after thermal treatment of their films at 140 °C for required period, specified on the Figures: f_{TBA} = 5.0 (a), 7.5 (b), and 10.0 mol. % (c).

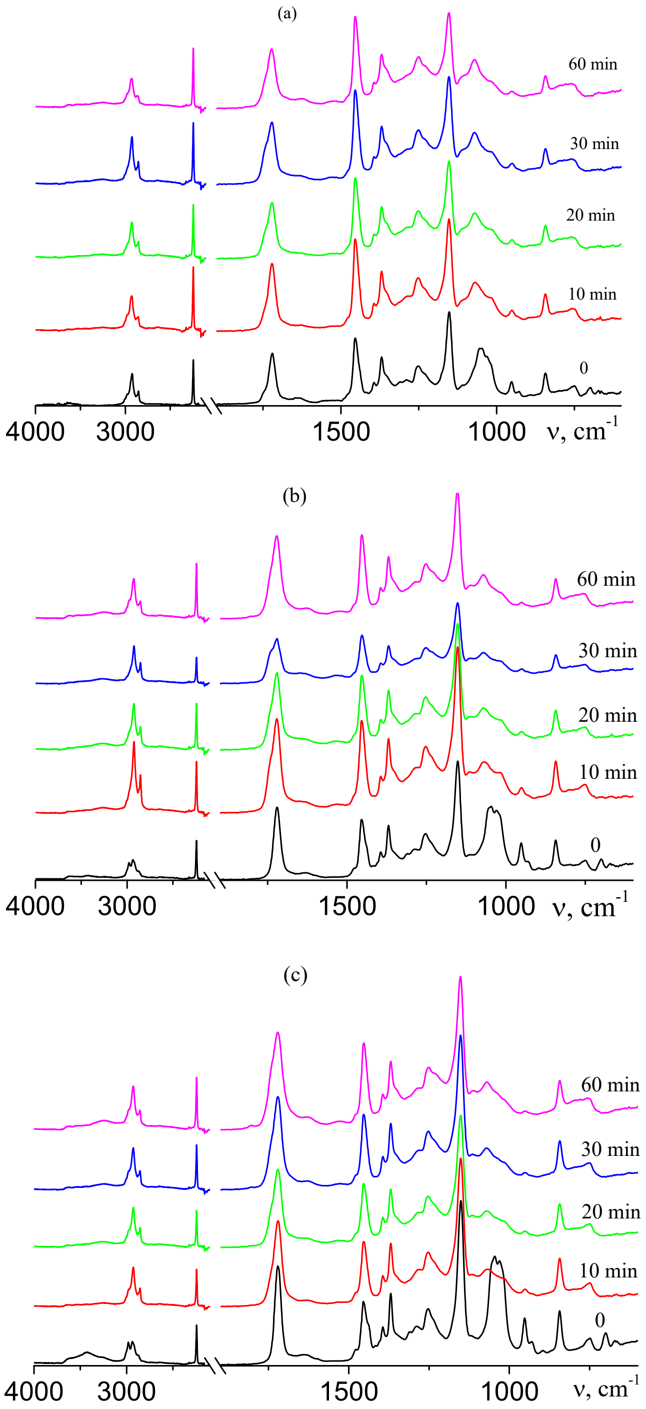

Figure S2. FTIR spectra of AN–TBA copolymers synthesized from different monomer feeds before and after thermal treatment of their films at 160 °C for required period, specified on the Figures: f_{TBA} = 5.0 (a), 7.5 (b), and 10.0 mol. % (c).

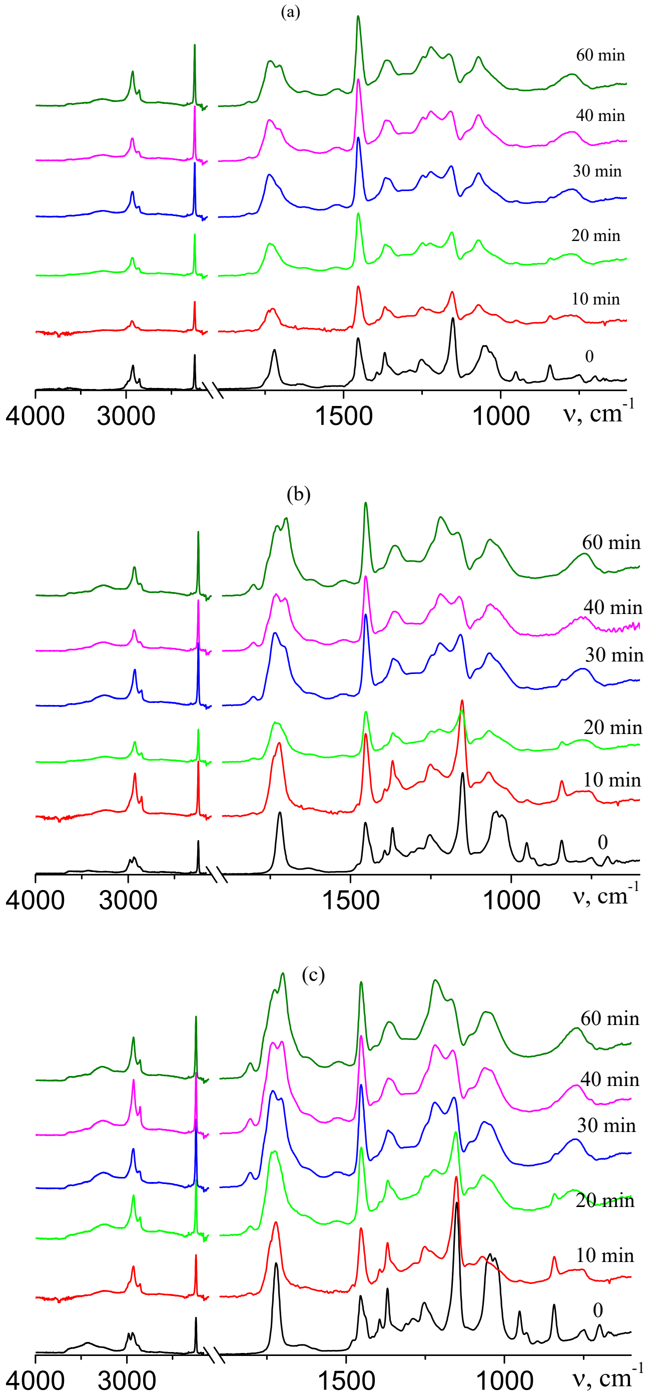

Figure S3. DSC thermograms recorded in argon of copolymers of AN–TBA with various content of TBA synthesized using various amounts of ME. (a) 1 mol. % TBA and 0.05 mol. % ME; heating rate 5 (1), 10 (2), and 20 ºC/min (3); (b) 2.5 mol. % TBA and 0.3 mol. % ME; heating rate 5 (1), 10 (2), and 20 ºC/min (3); (c) 5 mol. % TBA and 0.3 mol. % ME; heating rate 2.5 (1), 5 (2), 10 (3), and 20 ºC/min (4); (d) 10 mol. % TBA and 0.05 mol. % ME; heating rate 2.5 (1), 5 (2), and 10 ºC/min (3).

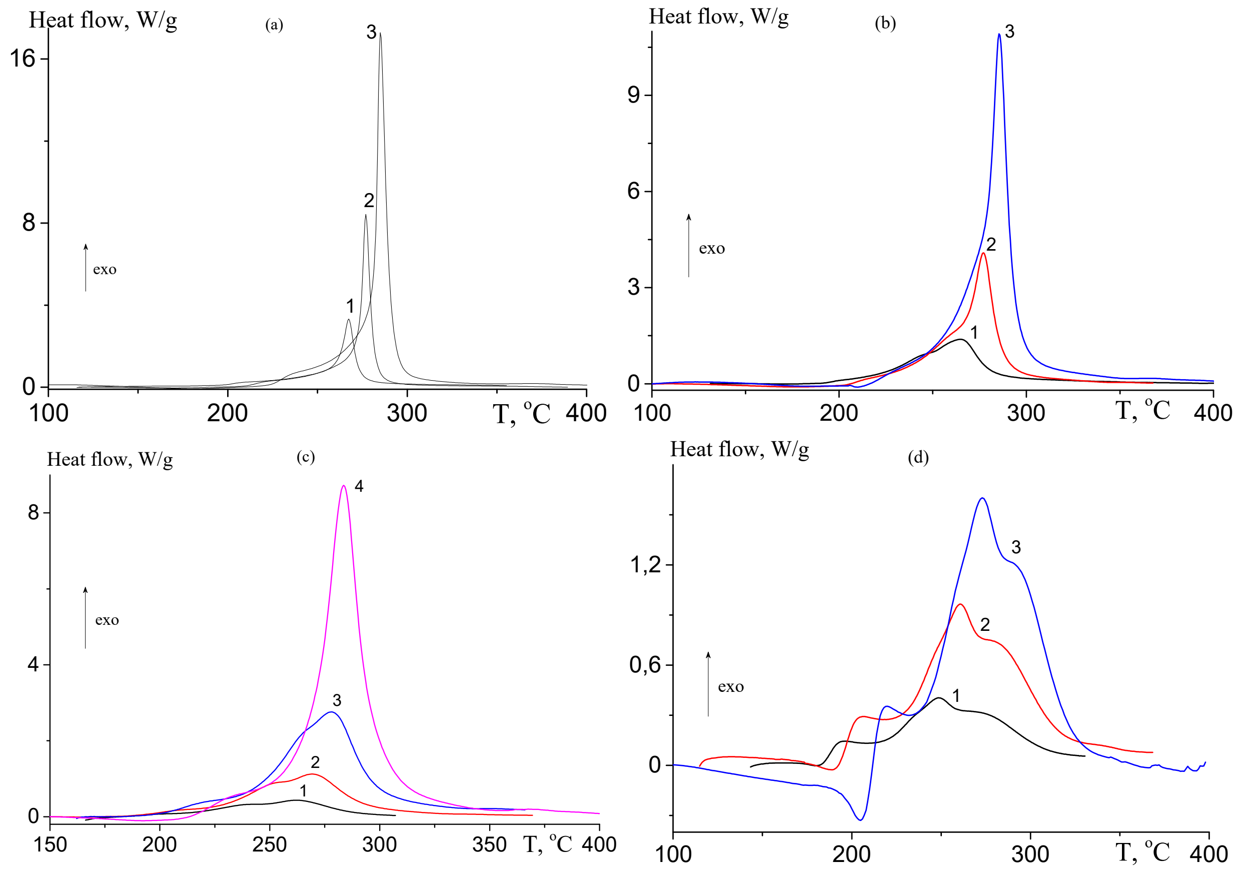

Figure S4. Dependences of $\ln(\varphi/T_p^2)$ on $1/T_p$ obtained from DSC thermograms of copolymers of AN and TBA at various heating rates; f_{TBA} = 1 (1), 2.5 (2, 3), 5 (4, 5), 7.5 (6, 7), and 10 mol. % (8, 9); peak II (2, 4, 6, 8), peak III (1, 3, 5, 7, 9).

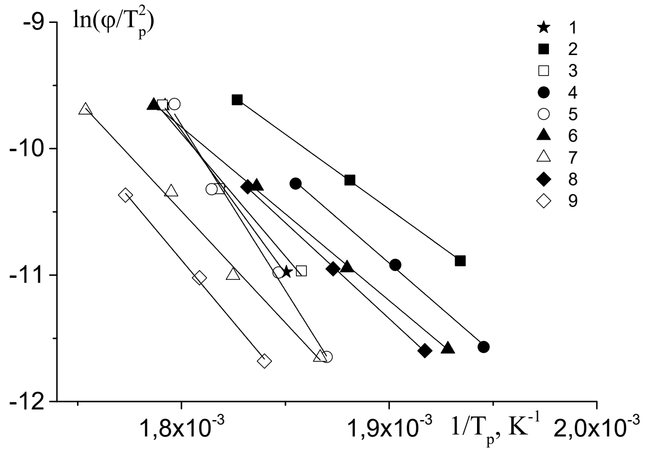

Figure S5. FTIR spectra of the films of AN–TBA copolymers subjected to thermal treatment at 200 (a, c, e) and 225 ºC (b, d, f) in argon at various times (indicated on Figure): f_{TBA} = 1 (a, b), 2.5 (c, d), and 5 mol. % (e, f).

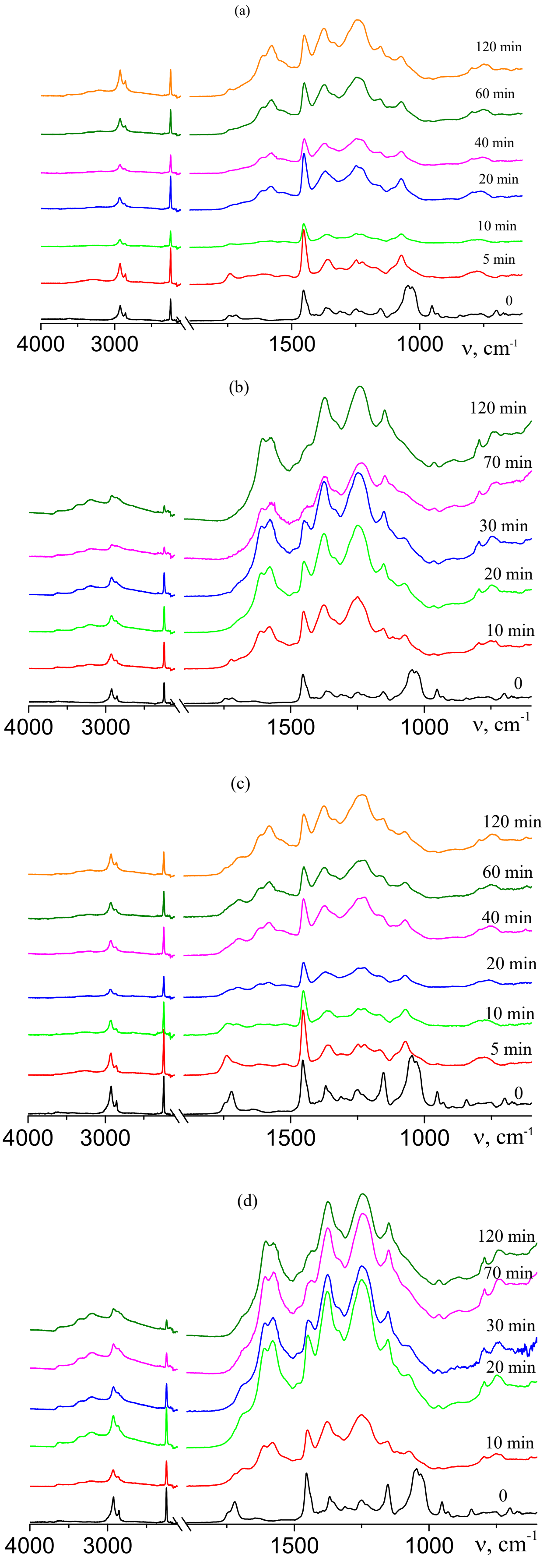

(e)

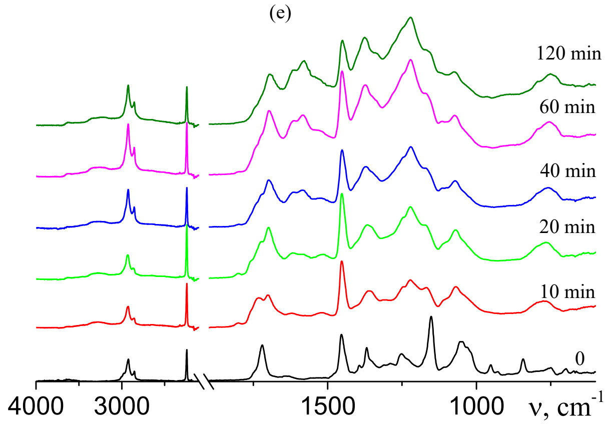
120 min
60 min
40 min
20 min
10 min
0
4000
3000
1500
1000
ν, cm-1

(f)

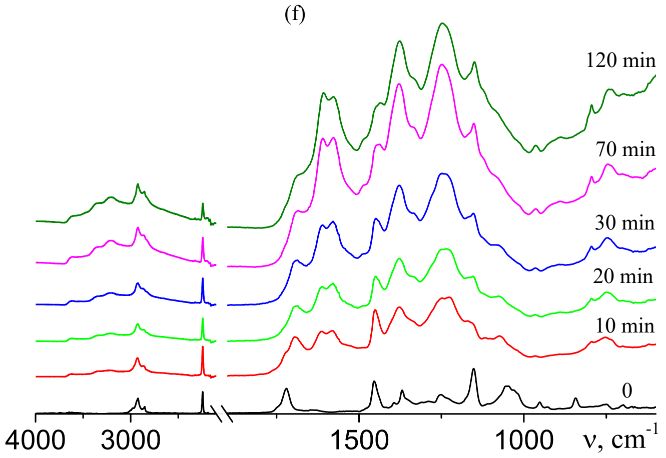
120 min
70 min
30 min
20 min
10 min
0
4000
3000
1500
1000
ν, cm-1

Figure S6. FTIR spectra of the films of AN–TBA copolymers subjected to thermal treatment at 200 (a, c, e) and 225 ºC (b, d, f) in air atmosphere at various times (indicated on Figure): f_{TBA} = 1 (a, b), 2.5 (c, d), and 5 mol. % (e, f).

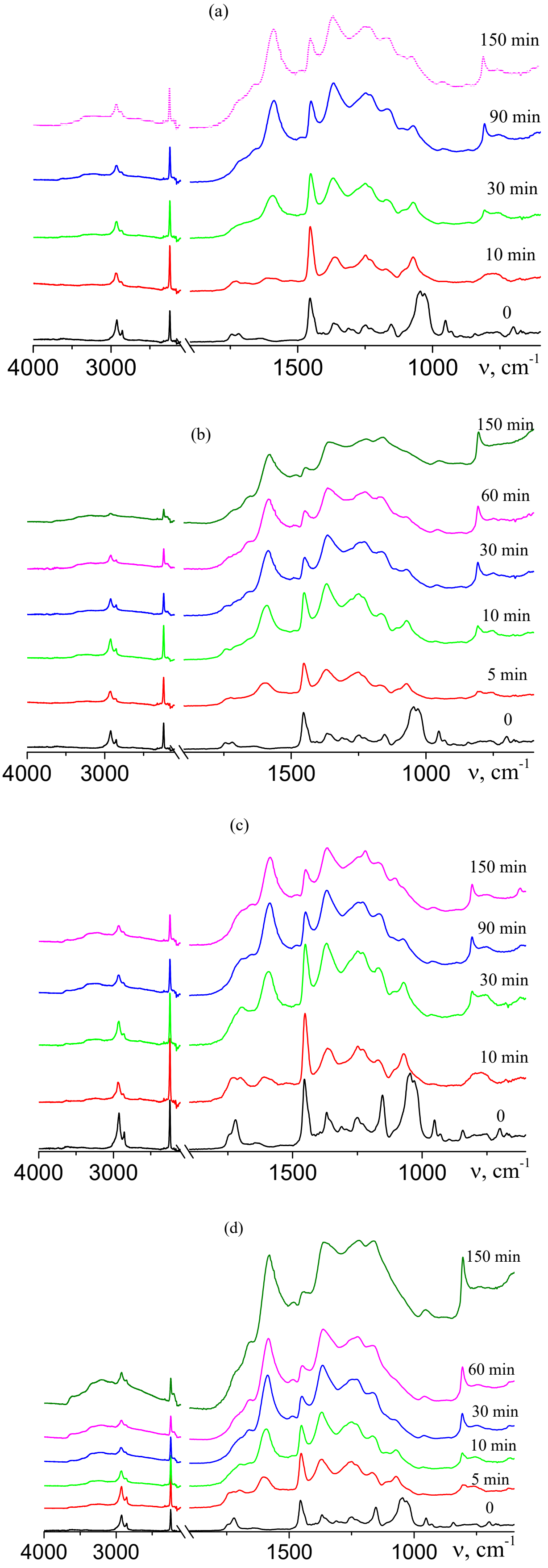

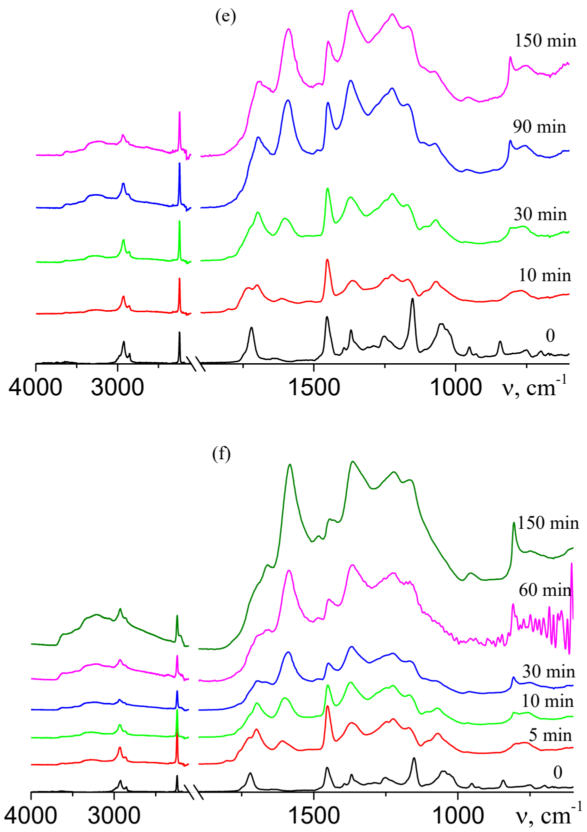
(e)
150 min
90 min
30 min
10 min
0
4000
3000
1500
1000
ν, cm-1
(f)
150 min
60 min
30 min
10 min
5 min
0
4000
3000
1500
1000
ν, cm-1

Figure S7. FTIR spectra of the films of AN–BA–TBA copolymers subjected to thermal treatment at 200 (a, c) and 225 ºC (b, d) in argon at various times (indicated on Figures): f_{TBA} = 1.0 (a, b) and 2.5 mol. % (c, d).

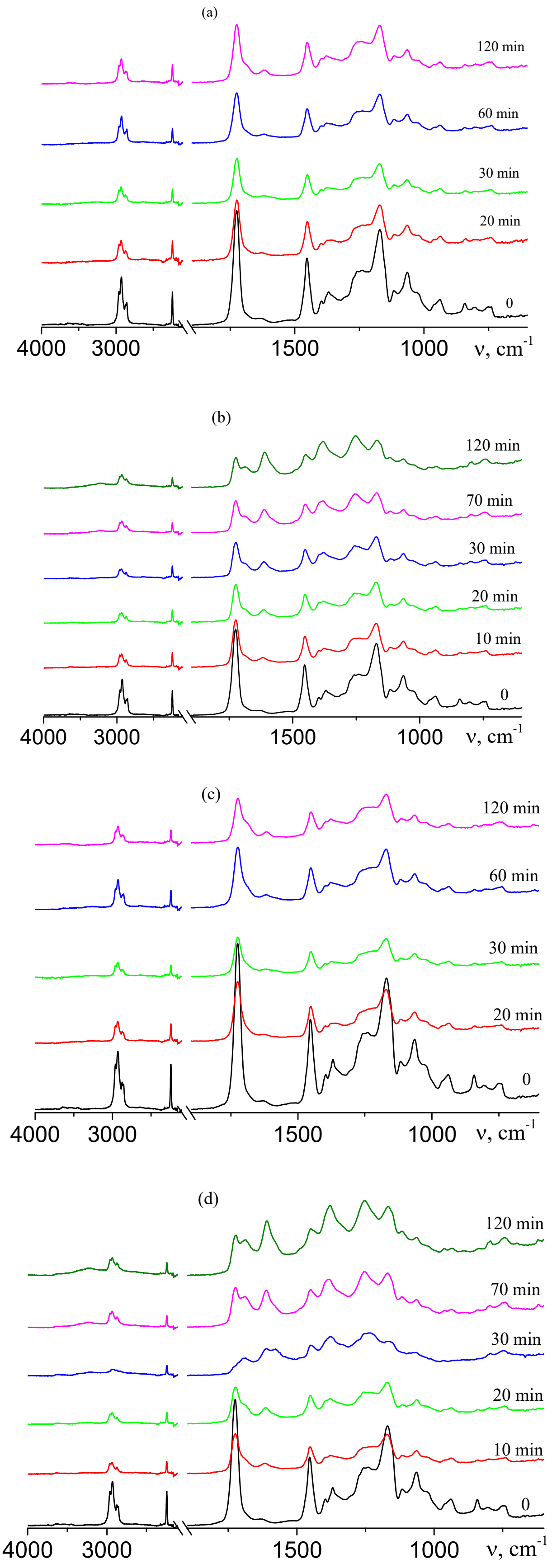

Figure S8. FTIR spectra of the films of AN–BA–TBA copolymers subjected to thermal treatment at 200 (a, c) and 225 ºC (b, d) in air atmosphere at various times (indicated on Figures): f_{TBA} = 1.0 (a, b) and 2.5 mol. % (c, d).

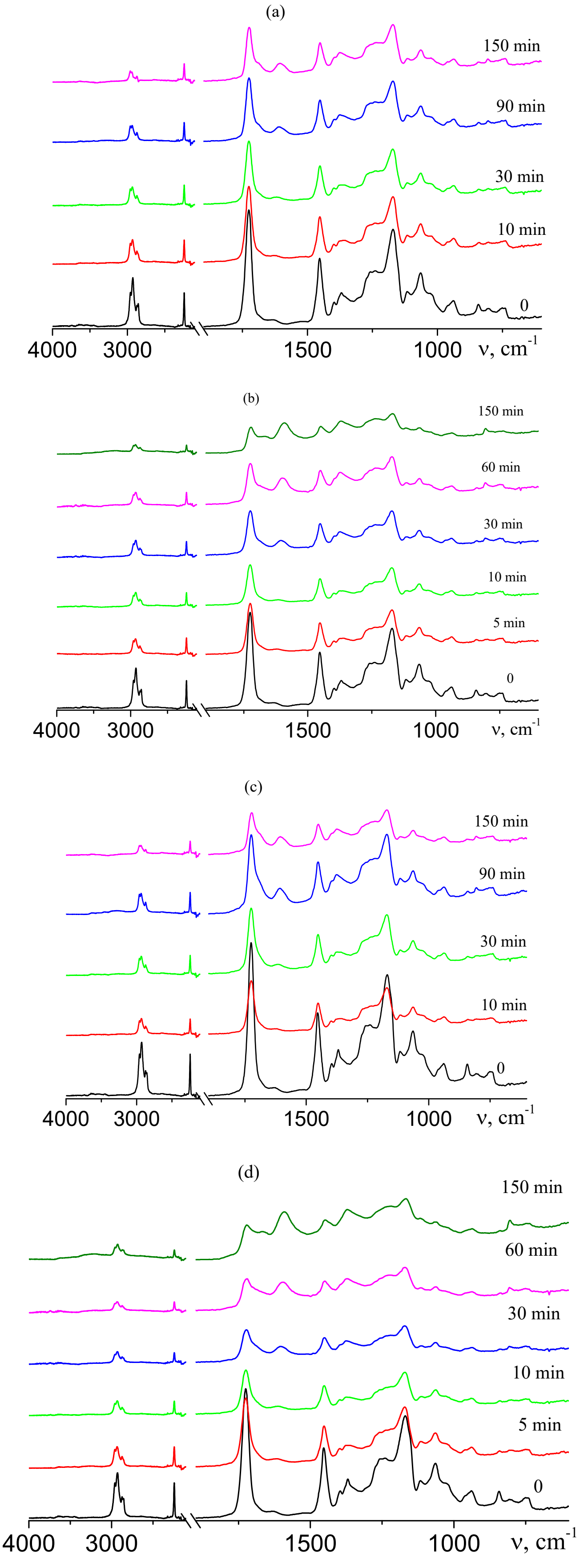

Supplement: Supplementary file 1 [file polymers-16-02833-s001.zip › polymers-3244321-supplementary.pdf]
